# Smart Nanocarriers for the Targeted Delivery of Therapeutic Nucleic Acid for Cancer Immunotherapy

**DOI:** 10.3390/pharmaceutics15061743

**Published:** 2023-06-15

**Authors:** Abu Baker, Jochen Lorch, David VanderWeele, Bin Zhang

**Affiliations:** Department of Medicine, Hematology/Oncology Division, Robert H. Lurie Comprehensive Cancer Center, Feinberg School of Medicine, Northwestern University, Chicago, IL 60611, USA

**Keywords:** cancer, immunotherapy, nucleic acids, nanoparticles, targeted delivery

## Abstract

A wide variety of therapeutic approaches and technologies for delivering therapeutic agents have been investigated for treating cancer. Recently, immunotherapy has achieved success in cancer treatment. Successful clinical results of immunotherapeutic approaches for cancer treatment were led by antibodies targeting immune checkpoints, and many have advanced through clinical trials and obtained FDA approval. A major opportunity remains for the development of nucleic acid technology for cancer immunotherapy in the form of cancer vaccines, adoptive T-cell therapies, and gene regulation. However, these therapeutic approaches face many challenges related to their delivery to target cells, including their in vivo decay, the limited uptake by target cells, the requirements for nuclear penetration (in some cases), and the damage caused to healthy cells. These barriers can be avoided and resolved by utilizing advanced smart nanocarriers (e.g., lipids, polymers, spherical nucleic acids, metallic nanoparticles) that enable the efficient and selective delivery of nucleic acids to the target cells and/or tissues. Here, we review studies that have developed nanoparticle-mediated cancer immunotherapy as a technology for cancer patients. Moreover, we also investigate the crosstalk between the function of nucleic acid therapeutics in cancer immunotherapy, and we discuss how nanoparticles can be functionalized and designed to target the delivery and thus improve the efficacy, toxicity, and stability of these therapeutics.

## 1. Introduction

Nucleic-acid-mediated immunotherapies have demonstrated efficacy in cancer treatments [1]. Different types of nucleic acids—mRNA, siRNA, miRNA, saRNA, gRNA, aptamer, and plasmid—can be used to modulate anti-tumor immunity. Nucleic acids can modify the expression of genes and thereby modulate the immune response to cancer or serve as an agonist for activating innate immune response pathways [2,3]. Research on immunomodulatory nucleic acids, ongoing for three decades, has produced several promising approaches for cancer immunotherapies and their development as candidates for clinical use [4]. Table 1 lists several major discoveries in the use of nucleic acids as modulators of the immune response to cancer (Table 1) [1]. Despite the potential for exploiting many classes of nucleic acids to develop cancer immunotherapies, many of them face major challenges regarding their delivery to target cells, including those of stability against nuclease-catalyzed degradation and uptake by targeted cells [5,6,7].

The innovation of smart nanocarriers that enable the use of nucleic acids for cancer immunotherapy indicates a major opportunity, particularly for protecting nucleic acids from degradation and enhancing targeted delivery. In contrast to other delivery systems, such as electroporation methods, gene guns, and sonoporation, the versatility and the unique magnetic, optical, chemical, and structural properties of nanoparticles offer numerous advantages to overcome biological barriers, including improved targeted nucleic acid delivery, appropriate intracellular processing and trafficking, a beneficial biodistribution and pharmacokinetic profile, and a controlled release through intelligent nanoparticle design [8,9]. Approaches to developing biocompatible nanoparticles as delivery vehicles for immunotherapeutic nucleic acids span a wide variety of materials (polypeptides, liposomes, chitosan, polyethyleneimine, proteins, and polymers) which have distinctive delivery advantages [10,11]. The properties of these nanoparticles can be engineered to obtain specific surface chemistries, sizes, physical properties, and material compositions for the delivery of nucleic acids. These nanoparticles are ultimately developed to promote delivery to target cells and to reduce off-target toxicity. Nucleic acids can either be encapsulated within or adsorbed onto the surfaces of nanoparticles with consequences on the shielding of nucleic acids from nuclease degradation and pharmacokinetic parameters (e.g., an increase in the circulation half-life) [12,13]. Additionally, nanoparticles can protect nucleic acids from acidic environments in solid tumors and release the nucleic acids in response to such stimuli [14]. The controlled release of nucleic acids by nanoparticles to cancer cells reduces the toxicity in healthy cells while increasing the delivery to cancer cells, which is advantageous for cancer therapy [15]. Here, in this review, we discuss different biocompatible nanoparticle vehicles for the delivery of nucleic acids with less toxicity, and their applications for cancer immunotherapy.

**Table 1:** Advancement of nucleic-acid-mediated therapeutics for immunotherapy.

Years	Milestones in Immunotherapy	Ref.
1995	Cytosine guanosine deoxynucleotide-mediated immune stimulation by bacterial DNA	[4]
2001	A clinical trial of mRNA with dendritic cells	[16]
2003	CTLA-4-specific aptamer used to manipulate the immune system	[17]
2005	miRNA cluster used to modulate tumor formation	[18]
2008	siRNA developed to trigger Toll-like receptor immune activation STING agonists identified for regulating innate immunity	[1] [3]
2016	A clinical trial of saRNA therapeutic CEPBA-51 in lipid nanoparticles for liver cancer	[19]
2018	A clinical trial of poly-ICLC, Hiltonol© combined with anti-PD-1 for the treatment of advanced solid cancer	[20]

## 2. Nucleic Acid Types and Structure

**Figure 1:**
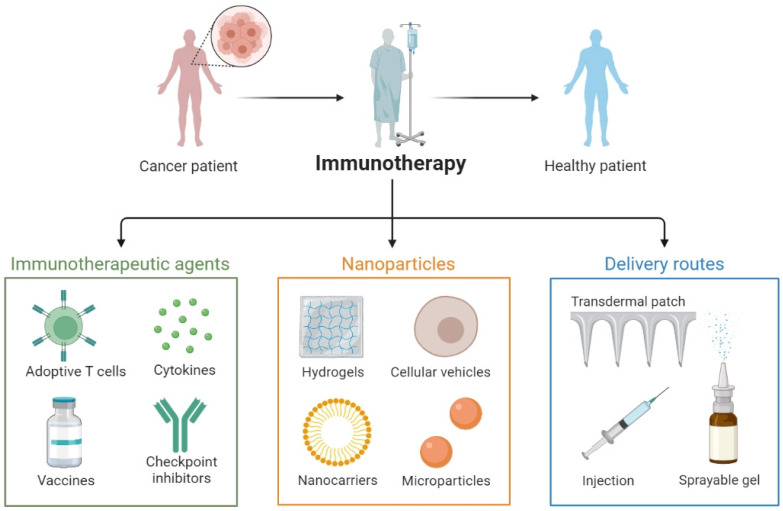
Schematic representation of universal therapeutics for cancer immunotherapy.

There are several types of nucleic-acid-mediated immunotherapeutics for the treatment of cancer, including immune stimulators (nucleic acids derived from pathogen-related molecular patterns) and plasmid, mRNA, aptamer, and immune modulators that act through gene regulatory mechanisms. Innate immunity is triggered by the recognition of immune stimulators and pathogen-associated molecular patterns (PAMPs) [21]. Furthermore, plasmids and mRNA can be used to encode and induce the biosynthesis of proteins and peptides which modulate anticancer immune responses (e.g., PD-L1 and STAT1 mRNA) [22,23,24]. In addition, nucleic acid tools for gene editing, suppressing, and activating systems can be engineered to enhance anticancer immunotherapeutic responses. Aptamers can trigger anticancer immune responses and promote the efficacy of immunotherapy [25]. Aptamers consist of single-stranded DNA or RNA oligonucleotides (ODNs) with a 3D folding that has a high specificity and affinity towards their targets [26]. Aptamers prevent alpha-toxin-induced cytolysis and activate TNF-α and IL-17 (AT-27, AT-33, AT-36, and AT-49) [27]. The target cells for various classes of nucleic acid immune modulators are broad, potentially including APCs, effector cells, and tumor cells. Approaches for delivering nucleic acid agents to these target cells need to address the need for biocompatibility and overcome the barriers encountered in therapeutic application (i.e., bioavailability, cellular uptake, and sub-cellular distribution). There are several components (active agents, delivery vehicles, and route of administration) for a wide variety of immunotherapies for cancer (Figure 1).

### 2.1. Circular RNA

**Figure 2:**
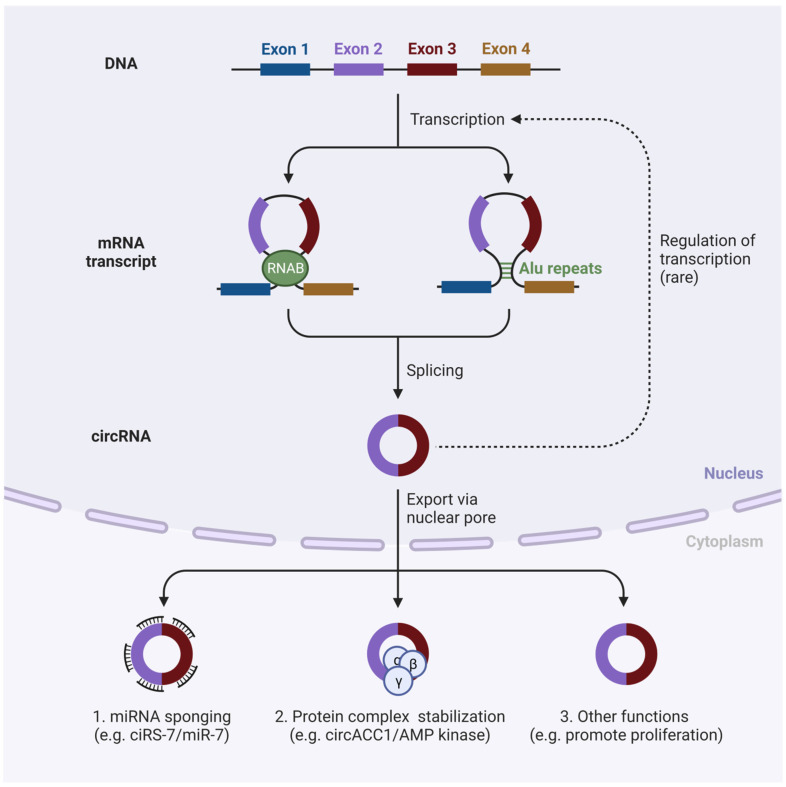
Biogenesis and function of circRNAs in cancer.

Circular RNA (circRNA), a novel non-coding RNA, is produced by the back-splicing of mRNA precursors. CircRNAs lack free 3’ and 5’ ends and have covalent closed-loop structures [28]. CircRNA is stable, acts as an effective therapeutic target, and is also over-expressed by expression plasmid and RNAi. CircRNAs have received little attention, and their biological regulation remains under investigation. CircRNAs can exert control over transcription and translation, can sequester and move proteins, and promote protein–protein interactions, among many other regulatory tasks [29,30,31]. CircRNA dysregulation has been linked to several illnesses, particularly malignancies, cardiovascular conditions, and neurological disorders (Figure 2) [32]. CircRNAs have the potential to be useful therapeutic targets since they are very stable and typically express themselves in tissue or in a cell-type-specific manner [33,34]. CircRNAs are often expressed using expression plasmid and knocked down by utilizing RNA interference (RNAi)-based techniques. The instability of siRNA, the lack of cell specificity, the limited intracellular penetration, the immune system activation, and other off-target effects of siRNA are just a few of its drawbacks [35]. These compounds can be delivered via exosomes or nanoparticles to enhance their immunogenicity, intracellular penetration, and stability [36]. Recently, circRNAs in particular cells were knocked down using the cre-lox method [37,38]. Numerous circRNAs have a variety of expression profiles in human malignancies. Strong evidence was recently presented that circRNAs function in cancer independent of their linear counterparts. To assess the significance of particular circRNAs in prostate cancer, high-throughput shRNA-based screening was carried out. CircRNAs were found to be crucial for cell proliferation in this investigation. These circRNAs may be used as therapeutic targets in the future. Below, we present a few instances of circRNAs that have been used as targets for various tumors (Table 2) [39].

### 2.2. mRNA

mRNA has recently sparked considerable interest in drug research, particularly for cancer immunotherapeutics [55]. mRNA is made up of a coding region (antigen translation) and a noncoding flank. When mRNA therapies are supplied to the cytoplasm, they are promptly translated, unlike DNA gene treatments, which require nuclear access. mRNA has been investigated for a variety of applications in cancer immunotherapy, in particular, therapeutic vaccination. Raising antigen-specific CD8^+^ T cell and CD4^+^ T cell populations is crucial in cancer immunotherapy. The modularity of mRNA allows for the simple integration of multi-epitope antigens and synergistic immunostimulatory signal peptides into a single mRNA vector for efficient induction of a wide range of antitumor immune responses [56]. Similarly, antigens capable of binding with human leukocyte antigen (HLA) can be simply added for immune regulation in the construction of human mRNA vaccines [57]. Aside from the synthetic subunit antigen mRNA vaccine, total tumor RNA has been explored for its ability to activate a broad range of tumor-specific antitumor immune responses for tumor immunotherapy [7]. Overall, mRNA vaccines hold substantial promise for cancer immunotherapy.

### 2.3. DNA

DNA-based cancer immunotherapy is a potent method for activating the immune system to combat cancer cells. Plasmids are used as the carrier of genetic material DNA. Generally, synthetic plasmids acquire more than one selective marker gene and a single synthetic polyclonal site sequence, which consists of more than one restriction enzyme recognition site [58]. The chimeric antigen receptor T cell (CAR-T cell) is one of the prominent applications of immunotherapeutic plasmids [59]. Cells are treated with plasmid DNA to elicit immune responses against the encoded antigen [60]. Early research using mice showed that plasmid DNA may trigger immune responses against cancer, influenza, and HIV-1 transgenic products, establishing DNA as a viable vaccination platform [61]. However, nucleic acids are degraded in the presence of nucleases and are quickly removed from circulation if no other carriers are designed [62]. Furthermore, without an exogenous transfection agent or delivery vehicle, negatively charged DNA often cannot pass the anionic cell membrane [62]. DNA must cross the nuclear membrane once inside the cells to enter the nucleus. Finally, it is essential to transfect DNA into the intended cells while minimizing off-target delivery [5].

One immunotherapeutic that is frequently explored is CpG ODN, which belongs to the PAMP family of nucleic acids. CpG ODN consists of short synthetic DNA with an unmethylated CpG motif [63]. In contrast to vertebrate genomes, where CpG dinucleotides are typically methylated, bacterial genomes are more likely to include unmethylated CpG DNA [64]. CpG activates immune cells via the Toll-like receptor-9 (TLR9) signaling pathway. This leads to the activation of downstream signaling cascades, including IRAK, TRAF6, NF-kB, and MAP kinases. Uptake and endosomal maturation are necessary for CpG DNA to activate the immune response, because TLR9 is located within the membrane of endosomes. Through TLR9 signaling pathways, CpG can activate NK cells, B cells, and DCs.

Double-stranded RNA (dsRNA) and cytosolic dsDNA are other PAMPs. Cytosolic dsDNA stimulates cGAS and proinflammatory immune responses. Cytosolic dsDNA can activate cGAS to synthesize 2′3′-cGAMP, which activates the STING signaling pathway to promote type-I IFN responses [65]. Different types of physiochemical methods are available to enhance the delivery of DNA such as electroporation methods, gene guns, and sonoporation for ex vivo use, but such methods cannot be used in vivo [66]. Nanoparticle-mediated delivery of DNA is a promising solution.

## 3. Nanoparticle-Mediated Nucleic Acid Delivery Systems for Immunotherapy

**Figure 3:**
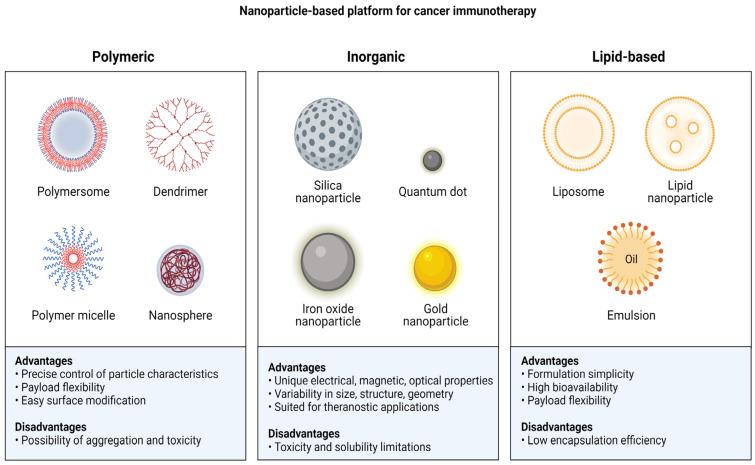
Nanoparticle-based platform for the delivery of nucleic acids.

Nanoparticle-mediated delivery for cancer immunotherapy can be categorized into two types based on their preparation: biologically synthesized nanoparticles and chemically synthesized nanoparticles. Biologically derived nanoparticles consist of biological components or cell derivative components, for example, exosomes and lipid-mediated nanoparticles, while chemically synthesized nanoparticles are categorized into polymers and inorganic nanoparticles (Figure 3). The following section describes these nanoparticle-mediated delivery systems. Nucleic acid molecules are shielded by nanoparticle delivery systems [67].

### 3.1. Lipid Nanoparticles

Lipids are biological components that are the building blocks of the biological membrane [68]. Liposomal nanoparticles have been approved by the FDA for clinical applications for cancer. Liposomal nanoparticles have been developed for site-targeted delivery (i.e., through conjugation with a targeting ligand) with a low toxicity and a high loading capacity due to the biological structure, tunable functionalization, and controlled release. They are thus attractive for use in delivering immune modulators. The properties and performance of lipid nanoparticles can be controlled by changes in the ratio of lipid constituents, surface chemistry, and method of synthesis; these variables influence the physicochemical nature of the lipid nanoparticles such as its size, shape, charge, and biological properties (i.e., the immune reaction, the efficiency of targeting, and the release of loaded ligands). Cationic lipid nanoparticles used in preclinical and clinical trials for gene and drug delivery often include amine head groups in the lipids [69]. The lipid nanoparticles’ surface charge helps in the internalization of the cells due to the positive charge and activation of the immune response [70]. pH-sensitive lipid nanoparticles have been used to enhance the targeted delivery of nanoparticles to subcellular compartments, particularly for the cytosolic delivery of immobilized immune modulators, leading to enhanced anticancer effects. The modification of the surface chemistry by cell-penetrating peptides (octaarginine and fusogenic peptides) has been described as an efficient strategy for inducing cross-presentation [71]. These approaches are in the preclinical stage, demonstrating the need to continue development for clinical translation.

#### 3.1.1. Lipid-Nanoparticle-Mediated circRNA Delivery

Accumulating evidence indicates the potential of circRNAs in the regulation of antitumor immune responses [72,73]. While circRNAs are under investigation as potential immunotherapeutic agents themselves, a major barrier to their application is the instability of circRNAs. Lipid nanoparticles can be used to encapsulate circRNAs to protect them from degradation, enhance cellular uptake and delivery via promoting the endosomal gateway for entry, and ultimately exert their antitumor function in vivo. CircRNA-loaded nanoparticles can be aimed at activating both innate and adaptive immune responses. For example, a novel ionizable lipid nanoparticle vaccine platform encapsulating the antigen-coding circRNA has been recently developed to exert the prolonged protein translation ability of circRNA and simultaneously create an innate immune stimulatory context, eventually leading to more potent cytotoxic antigen-specific T cell responses and a superior anti-tumor effect in multiple models of mouse tumors including “immune-desert” B16 orthotopic melanoma [74]. An additional study has reported that intratumoral delivery of circular mRNA encoding a mixture of cytokines by lipid nanoparticles facilitated intratumoral and systematic antitumor immune responses and enhanced anti-programmed cell death protein 1 (PD-1) antibody therapy in a syngeneic mouse tumor model [75]. Moreover, circRNA could serve as a potent adjuvant to trigger adaptive antitumor T cell immunity in the context of vaccination [76]. Nevertheless, there is still a long way to go in the clinical translation of nanoparticle-based circRNA immunotherapy for various malignancies.

#### 3.1.2. Lipid-Nanoparticle-Mediated mRNA Delivery

Many bioengineered nanoparticles are under development for delivering mRNA for immunotherapy, and some have advanced to clinical trials (e.g., the mRNA pancreatic cancer vaccine). Nanoparticles protect mRNA from nucleases and increase the internalization of mRNA into APCs, which increases the efficacy [77]. Lipid nanoparticles consist of different types of lipids (cationic, anionic, and neutral). Lipid nanoparticles contain ionizable lipids, meaning that these lipids are positively charged at low pHs (enabling RNA complexation) and neutral at physiological pH; they merge with the cell membrane to internalize which bypasses lysosomal degradation [78,79,80]. Nanoparticle uptake by the cell involves several highly regulated mechanisms such as phagocytosis, macropinocytosis, clathrin-mediated endocytosis, and caveolae, depending on physical properties of the nanoparticles such as size and shape, the chemical characteristics such as surface charge and modification, and the environmental conditions [79,81]. Cationic nanoparticles have been shown to efficiently deliver mRNA in vivo and elicit anti-tumor immune responses [82]. Cationic lipid head groups have a positive charge, whose main functions are to provide electrostatic interactions between the lipids and the negatively charged nucleic acid and to control the interaction of the nanomaterial with the lipid membranes of target cells. Positively charged mRNA-encapsulated lipid nanoparticle vaccines stimulate cellular and humoral immune responses in both mice and humans, which leads to the synthesis of antigen-specific IgG antibodies and activation of antigen-specific T-cell responses for cancer immunotherapy [83]. Moreover, the addition of polyethylene glycol (PEG) in lipid nanoparticles increases the stability and immobilization of nucleic acids but limits the uptake into cells [84]. Clinical trials of mRNA-based vaccines have begun recently for patients with melanoma and squamous cell carcinoma. Lipid nanoparticles with mRNA aimed at inducing immune responses against the antigens NY-ESO-1, MAGE324, A3, tyrosinases, and TPTE are in clinical trials [82]. Furthermore, lipid-nanoparticle-formulated nucleoside-modified mRNA vaccines encoding 20 patient-specific mutated neoepitopes are currently under clinical evaluation in combination with pembrolizumab in patients with solid cancers (NCT03313778 and NCT03897881). However, there are limitations to the number of currently clinically attempted antigens within mRNA, as true cancer-associated antigens encoding mRNAs represent only a very small fraction. The design space for mRNA has been expanded beyond therapeutic cancer vaccination using different nanoparticle formulations, delivery routes, and structural modifications. mRNA delivery by various nanoparticles is now employed for the most diverse immunotherapeutic approaches, including the equipping of immune cells with chimeric antigen receptors and the in vivo expression of immunomodulator proteins such as therapeutic antibodies, cytokines, or costimulatory molecules [85].

#### 3.1.3. Lipid-Nanoparticle-Mediated DNA Delivery

For the delivery of DNA, lipid nanoparticles are synthesized to prevent external hindrance. Delivery of plasmid-DNA-encoding costimulatory molecules and/or cytokines that stimulate tumor immunogenicity can be utilized efficiently by a nanoparticle because of its versatility. Indeed, a recent study has demonstrated the ability of a synthetic, biodegradable gene delivery nanoparticle platform to reprogram cancer cells in situ as tumor-associated antigen-presenting cells by inducing the coexpression of a costimulatory molecule (4-1BBL) and an immunostimulatory cytokine (IL-12) [86]. Researchers synthesized a DNA-encapsulated liposomal vaccine which encodes for the melanoma antigen (MART1) [87]. Liu et al. synthesized hydrophobic lipid nanoparticles with the help of DOPE for the delivery of CpG ODN along with the ovalbumin antigen. The acquired nanovaccines provide effective antigen stimulation which promotes the release of immune factors and leads to a boost in antitumor immunity to inhibit tumor growth [88]. Numerous types of nanoparticle-based DNA delivery systems are under evaluation in both preclinical and clinical settings for cancer immunotherapy [89,90,91].

### 3.2. Extracellular Vehicles

Extracellular vehicles (EV) are small particles which help in therapeutics and are generally synthesized by live cells. EVs are approximately 30 to 150 nm in size and found in different biological fluids [92,93]. Extracellular vehicles contain transmembrane markers, peptides, and specific proteins. EVs released from immune cells have unambiguous proteins and endosome-linked peptides, while EVs released from cancerous cells have an unambiguous tumor antigen. EVs are also now under investigation as delivery vehicles for immunotherapeutics. EVs are divided into immune-cell-derived extracellular vehicles and tumor-derived extracellular vehicles. The construction of synthetic EVs can address challenges associated with the need for sterility and scale manufacturing. Immune cells and dendritic cells derived from EVs have been reported to deliver MHC complexes such as MHC I and MCH II. CD80 and CD86 T cell stimulatory molecules and integrins act as adhesion molecules that additionally activate T cells [94,95].

Due to the unique tumor microenvironment and external treatment stimuli such as hypoxia and thermal stress, tumor-derived extracellular vesicles are typically more productive than immune-cell-derived extracellular vesicles [96]. According to reports, these aggressively secreted substances contain unique tumor antigens that dendritic cells take up to trigger potent tumor-specific cytotoxic T lymphocyte responses [97]. These extracellular functionalities of tumor derivatives have been used in several kinds of research to describe particular immunogenic responses for cancer immunotherapy. Additionally, nucleic acids or cytokines have been conjugated to extracellular vehicles to boost the tumor-derived extracellular vehicle’s efficiency in immunogenic activation, leading to the development of vaccines based on these extracellular vehicles. Future applications of tumor-derived extracellular vehicles as nanocarriers for cancer immunotherapy are hampered by the lack of clarity surrounding their precise impact and role on immunity. The therapeutic role of these extracellular vehicles for cancer immunotherapy needs to be assessed through research and safety evaluations.

### 3.3. Polymeric Nanoparticles

Different types of polymers are widely used for the synthesis of polymeric nanoparticles which are used for cancer immunotherapy [98]. These polymer-mediated nanoparticles can act as both adjuvants and delivery vehicles for immune-stimulating compounds when immune responses are stimulated to hinder the growth rate of tumors. Polymer-mediated nanoparticles are widely used as adjuvants in cancer immunotherapy due to their superior biocompatibility and solubility and high loading efficiency for immune-coupled components. Surprisingly, certain useful polymer-mediated nanoparticles are capable of stimulating immune responses against tumor cells. Polymer nanoparticles consisting of a cationic and responsive polymer such as poly (b-amino ester) have been examined to load ionic mRNA via an electrostatic interface, and these nanoparticles show enhanced transfection effectiveness and the curative effects of mRNA in vitro [56]. A bioactive polymer called inulin acetate (InAc) was used in conjunction with a poly lactic-co-glycolic acid (PLGA) as an associated delivery platform that could serve as a pathogen-incorporated vaccine delivery system [99]. The most frequent choice for polymeric nanoparticles in cancer immunotherapy is the PLGA nanoparticle [100]. PLGA nanoparticles have the self-capability to target dendritic cells and APCs. PLGA nanoparticles are also able to attach to tumor antigens and adjuvants. These biodegradable and biocompatible PLGA nanoparticles have been approved by FDA for delivery applications. Moreover, in vivo, research revealed that PLGA-loaded nanoparticles moved to the lymph nodes, where they activated dendritic cells and boosted CTL responses, which improved the efficacy of melanoma, bladder, and renal cell carcinoma. To transport more antigens to the targeted region and improve DC targeting, surface modification of PLGA nanoparticles was investigated. Although PLGA nanoparticles can increase immunity in preclinical models, their clinical performance still must be enhanced. Typically, an amphiphilic polymer’s hydrophobic moieties constitute the inner core of the structure, while hydrophilic residues form the outer covering [101]. Alternative nanocarrier substrates are required since many strong immune-stimulating drugs are protein based.

Hydrogel-mediated nanoparticles are particularly appealing for delivering hydrophobic compounds because of their exceptional capacity for hydrophobic molecules. Unfortunately, there are problems with current nano hydro gel platforms, especially concerning their unpredictable release characteristics. The physiological setting and inherent qualities of hydro gels prevent their further use in clinical research due to rupture release and short-term delivery caused by the expanded pore size and rapid degradation [102]. To enable construction with reliable and sustained delivery, improvements to better manage the cargo release from hydrogels are required.

### 3.4. Inorganic Nanoparticles

Cancer immunotherapy can make extensive use of inorganic nanoparticles by way of the precise control over the size, charge, and surface modifications [103]. They offer great efficiency and reproducibility for cancer vaccination techniques [104]. Inorganic nanoparticles have distinctive optical features that can be used for controlled tumor ablation with immunotherapy using nanoparticles [105].

Of all the inorganic nanoparticles, gold nanoparticles (AuNPs) have undergone extensive research for tumor immunotherapy. They are excellent delivery candidates to deliver tumor antigens and immune adjuvants to the targeted site due to their compliant surface chemistry and customizable shape [106]. Interaction of AuNPs with dendritic cells leads to triggered immune stimulant cytokine expression and downregulates immunosuppressive chemokines [107]. The elevated phagocytic features of dendritic cells and the improved maturation and triggering of T cell-linked immunological reactions are signs that AuNPs trigger dendritic cell activation [108]. AuNPs can serve as adjuvants when conjugated with CpG ODN (and other nucleic acids for immune response), in addition to their capacity as carriers of antigens to dendritic cells. Additionally, AuNPs can attract near-infrared radiation due to their unique optical properties, which generates heat for tumor ablation, a treatment method known as photothermal therapy. AuNPs induce tumor cell apoptosis and necrosis using photothermal therapy and appear to release tumor antigens and intrinsic immune adjuvants such as heat shock proteins (HSPs), which significantly stimulated immune activation [109].

Other metallic nanocomposites, such as silver and iron, have demonstrated tremendous activity against cancer immunotherapy in addition to the well-established AuNP systems. Metallic nanoparticles (MNPs) are used for therapeutic purposes in ongoing or finished clinical trials due to the above-mentioned intrinsic benefits. A well-known nanomedicine called CYT-6091 has been investigated in early clinical trials for cancer immunotherapy. It is made of AuNPs functionalized with thiolated PEG and recombinant human tumor necrosis factor-a (rhTNF-a) [110]. Advanced-stage cancer patients participated in phase I dosage escalation trials testing CYT-6091. According to the results, rhTNF formulated as CYT-6091 was given systemically, resulting in precise tumor targeting with no side effects.

Even with MNPs’ examination in ongoing clinical phase trials, their potential clinical applicability is debatable, mostly due to safety concerns such as the accumulation of AuNPs over time in the organs. After 10 months of research in dogs, AuNPs were found to be toxic; the results revealed pigmentation and accumulation in the liver, spleen, and lymph nodes [111]. In addition, the oxidation of DNA, lipids, and protein in cells is caused by ROS and free radical generation by AuNPs. This could change cell functions and even result in cell death [112].

### 3.5. Hybrid Nanoparticles

Hybrid nanoparticles can consist of both inorganic (e.g., metal ions, metal clusters, or particles, salts, oxides, sulfides, non-metallic elements, and their derivatives) and organic (e.g., organic groups or molecules, ligands, biomolecules, pharmaceutical substances, polymers) components to exploit the advantage of both components to enhance their biocompatibility and efficiency and reduce their toxicity. Hybrid nanoparticle synthesis is based on two strategies: barge and tanker. According to barge and tanker strategies, liposomes, micelles, polymers, and noble metals are encapsulated (barge) or accumulated at the surface (tanker) of nanoparticles [113]. Magnetic gold multifunctional hybrid nanoparticles have been developed, which consist of a gold nanoshell with a superparamagnetic iron oxide silica core, allowing MR imaging as well as photothermal therapy [114]. Efficient eradication of primary tumors and metastasis in mouse models can be achieved using PLGA nanoparticles co-encapsulated with hollow gold nanoshells (HAuNS, a photothermal agent) and an anti-PD-1 peptide (AUNP12) by blocking the PD-1/PD-L1 pathway and potentiating antitumor T cell responses [115]. Additional strategies include hybrid nanoparticles synthesized for the delivery of mRNA by using cationic lipids (e.g., DOTAP) and cationic biopolymers (e.g., protamine), which increases the transfection efficacy compared to lipid-mediated mRNA delivery and polymer-mediated mRNA delivery alone. Another hybrid nanoparticle strategy is dendrimer-mediated lipid nanoparticles, which consist of PEGylated BODIPY dyes that combine mRNA delivery and NIR imaging [116]. There are many other examples of hybrid nanoparticles, including PLGA lipid nanoparticles loaded with CRISPER/Cas9 plasmids, which consist of PLGA, lecithin, DSPE-PEG-cRGD, and DSPE-PEG-biotin [117].

### 3.6. Spherical Nucleic Acids (SNAs)

Spherical nucleic acids (SNA) are another class of smart nanoparticles that are synthesized by chemically modifying spherical cores with dense layers of single- or double-stranded DNA or RNA [118]. SNAs have generated a great deal of interest since Mirkin and colleagues initially reported them in 1996 because they enable a new approach for gene and medication delivery. The distribution of nucleic-acid-mediated therapeutics and the biological activity of the nucleic acid presented by the nanoparticle are enhanced. Recent developments in SNAs have accelerated both their potential for usage in clinical settings and their biomedical applications.

#### 3.6.1. Selection of an Appropriate Core for SNAs

Selecting an appropriate nanoparticle core is crucial for the effective delivery of nucleic acids utilizing SNAs. The forms, sizes, and biological profiles of the core–shell assembly are directly influenced by this. For organized DNA/RNA grafting on their surfaces, inorganic nanomaterials have been employed, such as iron, gold, silver, Pd, and Pt nanoparticles [119,120,121]. To create more biocompatible SNAs, organic nanomaterials such as proteins, polymers, and liposomes have been used [83,122,123,124]. The most popular SNA core materials are covered in this section, along with an explanation of how they were developed for dense nucleic acid coverings.

##### Inorganic Cores for SNA

Gold nanoparticles (AuNPs), which are the most characteristic noble metal nanoparticles, have been extensively used as the primary material in SNA synthesis. The size and form of the final SNA products can be directly controlled by producing AuNPs using simple synthetic procedures to generate a wide variety of particle diameters [125]. To create gold core SNAs, 13 nm AuNPs were capped with thiol-DNA in 1996. This process allowed for colloidal aggregation to occur in a controlled, thermally reversible mode [119]. The dense coating of DNA influenced the optical, electrical, and biological properties [126]. As a result of the surface area of AuNPs, multifunctional components can be presented densely, facilitating advanced drug/gene delivery for therapeutic applications. Silver nanoparticles (AgNP), in addition to gold, make excellent candidates for DNA grafting. Magnetic resonance imaging (MRI) and magnetic structure construction for electronic memory are just a few of the numerous uses enabled by densely functionalized iron oxide nanocrystals (Fe_3_O_4_ nanoparticles) with a surface DNA coating [127,128]. Metallic nanoparticles with hydrophobic capping agents, such as platinum, aluminum, palladium, copper, cobalt, and their combinations, can be used to make more forms of SNAs [129].

##### Organic Cores for SNA

Even though inorganic nanoparticles are most frequently employed to make SNAs, their propensity to concentrate in organs such as the liver and spleen poses possible long-term health complications [105,130]. ODNs are frequently washed out of cells, whereas the nanoparticle cores remain inside once SNAs are internalized by endosomes during incubation [131]. As a result, recently created biodegradable as well as biocompatible nanoparticles, including proteins, liposomes nanoparticles, and poly(lactic-co-glycolic acid) (PLGA), have created intriguing new opportunities for SNA functions in the biomedical field.

Liposome

Liposomal SNAs (LSNAs) can be made with simple starting materials that are easily accessible [132]. LSNAs are often created by attaching nucleic acids with a component with a hydrophobic head, such as cholesterol, to the phospholipid bilayers of liposomes. LSNAs are biocompatible and chemically adaptable. The heat stability of LSNAs is governed by its lipid components, which also influence its DNA loading, serum stability, cellular absorption, in vitro immune activation, and in vivo lymph node gathering [133]. The serum stability and blood circulation of LSNAs with increased phase changeover thermal lipids and secured liposome scaffolds are enhanced. The stability of the overall nanostructure as well as the rate at which ODNs are released from the liposome core are impacted by the DNA shell’s affinity. Compared to cholesterol-based LSNAs, diacylglycerol lipid tails result in higher affinity LSNAs with a two-fold boost in ODN loading, a 20-fold increase in circulation half-life, and quicker cellular internalization with more powerful immune establishment [122]. In animals, LSNAs do not instantly leave the bloodstream; instead, they are internalized via the mononuclear phagocyte system, which is used to effectively activate immune cells such as dendritic cells (DCs) and macrophages [134]. When compared to free ODN, CpG-embedded LSNAs have an immunological potency about 80 times higher, making them effective cancer immunotherapeutic and liposome-based immune stimulants [131,135]. To enhance their anticancer efficacy, LSNA-conjugated tumor-related antigens alter the kinetics of the presentation of antigen and the assembly of stimulatory markers [136]. The spontaneous adsorption of DNA on the surface of liposomal nanoparticles influences the delivery of LSNAs in vivo; LSNAs conjugated with DNA by diacylglycerol lipid tails show significant DNA delivery to the kidney. In both cases, high accumulation is observed in the spleen, and cholesterol-based LSNAs demonstrate excellent DNA transport to the lung [137]. As a result of this, LSNAs can transport medicines and nucleic acids to various important organs simultaneously. The biological outcome of LSNAs, however, remains under investigation. Immunostimulatory liposome spherical nucleic acids have been adapted for cancer immunotherapy in both preclinical and clinical studies [131]. These structures were composed of CpG ODNs as an adjuvant along with different types of peptide antigens on the surface and inside the core [138]. The arrangement of these antigens on the surface occurs via hybridization. The hybridization model elevates the delivery efficacy. Vaccination with this hybridization model is more efficient in triggering antitumor T cell immune responses than other models [131].

Protein

A novel kind of SNAs are protein SNAs (Prot-SNAs), which are made up of a protein core and a thick ODN crust. They are capable of entering cells efficiently. To evaluate both their intracellular transport efficiency and catalytic performance simultaneously, Mirkin et al. immobilized ODNs onto the homotetrameric enzyme (b-galactosidase). ProSNA galactosidase demonstrated up to a 280-fold higher cellular uptake than b-galactosidase and decreased the enzyme optimizing concentration by up to 100 pM [123]. Pro-SNAs led to a seven-fold elevation in the cellular uptake when conjugated with ODNs using hexamethylene glycol linkers while retaining their enzymatic activity in vitro. Additionally, the production of Pro-SNAs from G-quadruplex resulted in a four-fold increase in the cellular uptake. Prot-SNAs display extended circulation and increased accumulation in important organs (such as the lung, kidney, and spleen) when utilized in animals, regardless of the order in which they are administered. They also preserve enzymatic functions [139]. The most recently reported cross-linking technique involves assembling a single Prot-SNA with a lactate oxidase core into nanoscale particles (referred to as cross-linked SNA, or X-SNA), which improves the intracellular Prot-SNA signal-to-noise ratio and cellular delivery efficiency [140]. Prot-SNA is anticipated to be very promising in protein-based therapeutic and diagnostic applications, including immunotherapy and enzyme substitute treatments [141].

## 4. Nucleic Acid Nanoparticles in the Cancer Immunity Cycle

**Figure 4:**
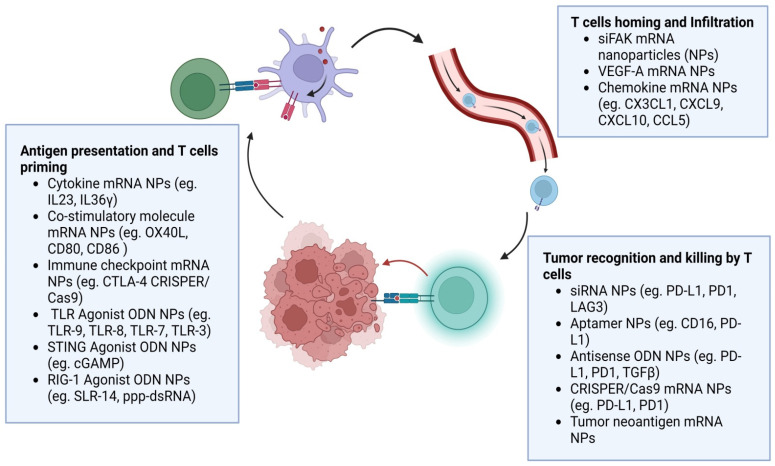
Immune modulatory nucleic acid nanoparticles in the cancer immunity cycle [153,154].

Nucleic acids can have an important role in cancer immunotherapy due to the multiple ways in which they can modulate the immune cell activity (activation of innate immunity, gene silencing, and translation of antigen and immunostimulatory proteins). Figure 4 shows multiple types of target cells within the tumor immune microenvironment and the potential for nanocarrier-enhanced modulation of activity through nucleic acid agents [142]. The PAMP class of immunomodulatory nucleic acids are recognized by TLRs on the membrane of endosomes and cGAS in the cytosol, which leads to the production of type-I IFN and an enhancement in the antitumor immune response by cytokines [143]. mRNA-molecule-encoding proteins can drive an RNA-mediated immune response by inducing the translation of proteins [144]. Gene regulatory nucleic acids, such as siRNA [145] gene activating nucleic acids, antisense ODN [146], and gene-editing nucleic acids, can regulate immune-related genes for the activation of anticancer immune responses. Aptamer agonists enhance immune-based molecular targets to endorse the anticancer immune response [147].

Nanoparticles can enhance the transport of cargo and deliver nucleic acids, directly targeting immune checkpoint molecules (e.g., CTLA-4 and PD-1/PD-L1) in a controlled way to extend their therapeutic efficacy. Indeed, emerging research has demonstrated that delivery methods based on nanoparticles enhance the concentration and retention of nucleic acids associated with immune checkpoint blockades in target immune cells and/or tumor tissues. For instance, liposome nanoparticles encapsulated with antisense DNA have been investigated for PD-L1 inhibition. In colon cancer, PD-L1 silencing was observed by SNA, in which both the surface and total PD-L1 expression were decreased, providing a promising path for cancer immunotherapy [148]. The targeted delivery of nucleic-acid-based antigens by nanoparticles is protected from nucleases, enhances their drainage to the lymph nodes, and decreases autoimmune toxicity. In most studies, intra-tumoral delivery of mRNA for co-stimulatory molecules and cytokines is accomplished by lipid-mediated nanoparticles, but they were also administered in a saline solution which facilitates translation to clinical applications. This method focuses on short-term local delivery of cytokines which trigger an effective anti-tumor response at the local tumor site (in situ vaccination). The anticancer efficacy of cytokine mRNA alone and along with PD-1 inhibitors is boosted. The extracellular matrix rigidity is reduced by siFAK+CRISPER-PD-L1 nanoparticles, which also disrupt PD-L1 expression by gene editing, in turn leading to a decline in tumor growth [149]. The anticancer activity of injected and non-injected tumor sites and stimulation of tumor-specific memory T cell activity were also determined. The use of site-directed intra-tumoral lipid-nanoparticle-encapsulated mRNA led to the translation of numerous cytokines (IL-23 and IL-36γ) and T cell activation agonist OX40L [150,151]. Nanoparticles have an enormous potential for modulating anti-tumor immune responses and cancer immunotherapy [152].

## 5. Challenges for Nucleic-Acid-Mediated Nanotherapeutics

Different types of nucleic-acid-related therapeutics have been approved by the FDA for various diseases; these do not include nucleic acids for cancer immunotherapy. However, even after three decades of research, nucleic acids face numerous physiological barriers after being given systemically or locally [155]. The pharmacokinetic and pharmaceutical features, such as the maximum time in blood circulation, the accumulation in tissues, the cellular uptake, and escaping endosomes, have been analyzed during the development of technologies for targeted delivery of nucleic acid therapy [156]. Recently, the lyophilization technique was introduced to improve the stability, storage, and transportation of lipid-based nanoparticles, which are prone to become unstable and aggregate in solution [157].

## 6. Conclusions and Future Outlook

In this review, we have described the advantages and disadvantages of different types of smart nanoparticles for delivering cancer immunotherapy. Nanoparticles can shield nucleic acids from deterioration and excretion. Work is still ongoing to improve the stability and cellular internalization and protect them from lysosomal degradation. Recent advancements in modification techniques make nanoparticles an excellent and mature nucleic acid delivery technology, with advances in production at scale for certain classes of nanoparticles. In addition to cancer therapies, traditional treatments such as chemotherapy and immunotherapy to treat tumors, hereditary illnesses, infections, and immunological deficiencies have advanced thanks to the widespread use of nanoparticles.

Many obstacles still exist despite success in numerous preclinical research and clinical situations. There are, however, safety concerns; for instance, some elements in nanoparticles may irritate the skin, produce inflammation, and trigger the body’s defense mechanisms. The limited circulation duration of the supplied nanoparticles and the potential cytotoxicity are additional difficulties that require careful consideration. More fundamental research is necessary to address these concerns.

Among the other nanoparticle platforms that have been developed, lipid nanoparticles are the most advanced because they are the least toxic for in vivo delivery of nucleic acids, and the use of PEG in lipid nanoparticle synthesis increases the half-life of the nanoparticles. The manufacturing of lipid nanoparticles can be easily scaled up with a lower investment. Their long-term stability is an issue, but it is extended by using an appropriate storage temperature. Nanoparticle-based nucleic acid delivery platforms will become more promising and advanced in upcoming clinical applications as the understanding of nanoparticles for nucleic acid delivery is strengthened.

## Figures and Tables

**Table 2:** CircRNA deregulation that has been targeted for cancer therapy.

CircRNA	Up Regulate	Down Regulate	Types of Cancer	Ref.
Circ-AGFG1	✓		Triple-negative breast cancer (TNBC)	[40]
Circ-HER2	✓			[41]
Circ-TADA2A-E6		✓		[42]
Has-circ-0025202		✓	Breast cancer (BC)	[43]
Circ-Dnmt1	✓			[44]
Circβ-catenin	✓		Hepatocellular carcinoma (HCC)	[45]
Circ-RNA-104718	✓			[46]
Circ-TRIM33-12		✓		[47]
Circ-RNA 100146	✓		Non-small cell lung cancer (NSCLC)	[48]
Circ-PTPRA		✓		[49]
Circ-CACTIN	✓		Gastric cancer (GC)	[50]
Circ-PSMC3		✓		[51]
Circ-HuR		✓		[52]
Circ-CUX1	✓		Neuroblastoma (NB)	[53]
Circ-LONP2	✓		Colorectal cancer (CC)	[54]

## Data Availability

Not applicable.
